# Effects of Muscle Function and Limb Loading Asymmetries on Gait and Balance in People With Multiple Sclerosis

**DOI:** 10.3389/fphys.2018.00531

**Published:** 2018-05-15

**Authors:** Thorsten Rudroff, Felix Proessl

**Affiliations:** ^1^Department of Health and Exercise Science, Colorado State University, Fort Collins, CO, United States; ^2^Department of Radiology, University of Colorado School of Medicine, Aurora, CO, United States

**Keywords:** multiple sclerosis, muscle function, asymmetry, limb loading asymmetry, gait, balance

## Abstract

People with MS (PwMS) often have a more- and less-affected side of the body which results in a variety of asymmetries, including measures of power, strength, muscle activity, and limb loading. Though many studies have identified asymmetries, their impact on gait and balance in PwMS is currently unclear. In this mini-review we first summarize previous findings of asymmetries in muscle function and limb loading and their impact on gait and balance in PwMS. We then provide potential explanations for this lack of consistency in the current literature, and propose study guidelines to improve future lower limb asymmetry studies. Making use of a unified approach to study lower limb asymmetry may then provide more clarity regarding their impact on mobility, specifically gait and balance, in PwMS.

## Introduction

An estimated 2.3 million people worldwide are currently diagnosed with multiple sclerosis (MS) (Browne et al., 2014), which is an inflammatory autoimmune disease of the central nervous system (CNS) (Noseworthy et al., 2000) manifested by a demyelination and neurodegeneration of the brain and spinal cord (Chaudhuri, 2013; Kindred et al., 2014). As a result, the travel of information between the CNS and the periphery is often slowed or blocked, leading to various symptoms depending on the location of the damage (Matthews, 1998; Schmierer et al., 2000).

An early symptom of MS is weakness on one side of the body, particularly in the lower limbs (White and Dressendorfer, 2005; Larson et al., 2014). Due to this unilateral weakness, people with MS (PwMS often have a more- and less-affected side of the body which results in a variety of asymmetries, including measures of power, strength, muscle activity, and limb loading (Chung et al., 2008; Van Emmerik et al., 2010; Kalron et al., 2011; Sandroff et al., 2013; Rudroff et al., 2014; Ketelhut et al., 2015; Kalron, 2016). Though many studies have identified asymmetries, their impact on gait and balance in PwMS is currently unclear. However, it is well established that impairments in mobility, specifically, gait and balance, can significantly increase the indirect costs of living and negatively impact quality of life and independence in PwMS (Larocca, 2011; Coleman et al., 2013). Nonetheless, to the best of our knowledge, there is currently no review summarizing the current state of the asymmetry literature in MS. A better understanding of the influence of muscle function and limb loading asymmetry on gait and balance may thus be a potential way for physical therapists to improve the design of rehabilitation programs of PwMS and could result in improvements of quality of life in these patients.

As a result, this mini-review aims to summarize previous findings of asymmetries in muscle function and limb loading and their impact on gait and balance in PwMS, provide potential explanations for the lack of consistency in the current findings, and propose study guidelines to improve future lower limb asymmetry studies to help physical therapists in the decision-making regarding treatment of asymmetries. Studies included in this mini-review were selected based on the following two inclusion criteria: (1) sample population being PwMS and (2) objective determination of asymmetry between sides of the body to investigate their influence on gait and balance (**Table 1**).

**Table 1:** Studies investigating the effects of asymmetrical muscle function and limb loading on gait and balance in People with MS (PwMS).

Study (author, year)	Study details/outcome measures	Main outcomes
Chung et al., 2008	*N* = 24 (12 Controls), no MS type reported, Peak knee extensor (KE), dorsiflexor isometric torque and power asymmetry, T25FW, limb loading asymmetry, fatigue, balance.	KE power was lower and KE power was greater in PwMS than in controls.
Kalron et al., 2011	*N* = 52 (CIS), EDSS 1.7 (SD = 1.3). Peak isometric strength (KE, KF, PF, DF) lower limb muscle fatigue, spatiotemporal gait parameters.	PwMS showed greater ankle muscle torque asymmetries.
Kalron, 2016	*N* = 402 (RRMS 389), Fallers/non-fallers. Vertical ground reaction forces (GRF).	GRF symmetry index score of the total sample was 3.7 (SD = 3.1). No significant correlations between GRF and fallers/non-fallers.
Sandroff et al., 2013	*N* = 31 (31 Controls), no MS type reported, VO_2_ peak, muscular strength (asymmetry between knee muscles), balance, T25FW, 6MWT	Knee extensor asymmetry was associated with walking performance and gait in PwMS. Lower limb strength asymmetries, but not balance, explained variance in walking performance and gait variables in PwMS and controls.
Ketelhut et al., 2015	*N* = 8 (RRMS) (8 Controls), PET/CT imaging for core muscle activity asymmetry during walking based on glucose uptake.	Within the MS group, side differences in activity were identified in the lateral flexor group, the external and internal obliques, and the rectus abdominis, with the less-affected side being activated more.
Van Emmerik et al., 2010	*N* = 12 (6 RRMS) (12 Controls), Balance under normal and restricted vision, limb loading asymmetry during quit standing and max. lean and reach tasks.	PwMS displayed greater loading asymmetry and limiting vision increased loading asymmetry during quit standing and postural instability during backward lean.
Rudroff et al., 2014	*N* = 8 (RRMS) (8 Controls), PET/CT imaging for muscle activity asymmetries based on glucose uptake in leg muscles of mildly disable PwMS.	Glucose uptake differences occur between the weaker and stronger legs of PwMS, specifically in the knee flexor group.
Larson et al., 2013	*N* = 8 (RRMS) (7 Controls), Bilateral differences in leg muscle strength and metabolism during exercise were quantified.	PwMS exhibited significantly greater asymmetry for strength, oxygen uptake, and workload than controls.
Proessl et al., 2018	*N* = 19 (RRMS), Leg strength symmetries, 6MWT, DWI_6-1_.	The magnitude of leg strength asymmetry did not correlate with walking ability, fatigability and fatigue.

*6MWT, 6-min walk test; CIS, clinically isolated syndrome (early onset of MS); DWI_6-1_, distance walked index; EDSS, expanded disability status scale; FSS, fatigue severity scale; MS, multiple sclerosis; PET/CT, positron emission tomography/computed tomography; PwMS, people with multiple sclerosis; RRMS, relapsing remitting multiple sclerosis; SD, standard deviation; T25FW, timed 25 foot walk test; VO2 Peak, peak oxygen consumption.*

## Muscle Function Asymmetries

Muscle function asymmetries have been repeatedly found among PwMS and include measures of strength, torque, oxidative capacity or glucose uptake. Sandroff et al. (2013) and Proessl et al. (2018) found asymmetries in knee extensor strength, while Rudroff et al. (2014) found asymmetric strength in the knee flexors but not in the knee extensors or dorsiflexors in PwMS. Another study by Kalron et al. (2011) measured peak isometric torque (PIT) and isometric fatigue index (FI) of the knee extensors, flexors, ankle dorsiflexors, and plantar flexors with an isokinetic dynamometer. FI was measured by performing 30 sec sustained MVCs. The force-time curve was used for FI calculation. The PIT asymmetry scores indicated greater asymmetry of plantar flexor and dorsi flexor muscle torque in PwMS than healthy subjects, with the knee extensors and flexors trending toward significance. No differences were found in any of the FI asymmetry scores.

Traditionally, electromyography (EMG) has been used to investigate muscle activity. However, due to the limitations of EMG (Farina et al., 2004) the detection of asymmetric muscle activity, especially in mildly disabled PwMS, is challenging. As suggested by Rudroff et al. (2015, 2017) positron emission tomography (PET) with [^18^F]-fluoro-deoxy-glucose ([^18^F]-FDG) as a glucose analog can be used to monitor cumulative muscle activity during exercise. In an FDG-PET study Rudroff et al. (2014) found that mildly disabled PwMS had greater FDG uptake in the knee and hip flexor muscles than healthy controls after 15 min treadmill walking at a self-selected speed. Furthermore, FDG uptake was significantly lower in the weaker knee flexors compared to the stronger side in PwMS, indicating greater metabolic cost during walking that may contribute to greater performance fatigability and impaired walking.

Postural and trunk control are also important during walking and PwMS often show impairments in these features during walking (Lanzetta et al., 2004). The activation of the deep core muscles of PwMS after treadmill walking for 15 min was measured with FDG-PET by Ketelhut et al. (2015). PwMS showed side differences in muscle activity, indicated by FDG uptake, in the lateral flexor group, with the less-affected side being activated more. The authors therefore suggested a potential compensatory mechanism that PwMS may use during walking to maintain balance and posture.

Another way to get further insight into asymmetric muscle function is to investigate oxidative capacity (VO_2_ peak). Larson et al. (2013), for instance, found that during unilateral incremental cycling, the strong leg performed at relatively higher capacity than the weaker leg, as indicated by higher VO_2_ peak, in PwMS.

While these findings included a variety of asymmetries in muscle function with a range of 10–38% in mildly disabled PwMS, altogether, almost all studies suggested that asymmetries in muscle function likely result in increased muscle energy cost, early fatigability, postural instability, or walking impairments.

## Limb Loading Asymmetries

Although limb loading asymmetries may impair postural stability and may increase the risk of falls in PwMS, only three studies addressed this important topic in PwMS (Chung et al., 2008; Van Emmerik et al., 2010; Kalron, 2016) and the evidence appears contradicting. Chung et al. (2008) used the center of pressure (CoP) as a measure of postural sway. CoP is representative of ground reaction force (GRF) and the trajectory of the center of mass. GRF is the force exerted by the ground the object is in contact with and provides indirect information about internal joint loading as peak GRF coincides with the timing of peak loads on the femur and hip joint during gait. Assessing bilateral distribution of body mass during quiet stance, Chung et al. (2008) found a greater limb loading asymmetry score in PwMS than in controls.

Another study by Van Emmerik et al. (2010) found that, compared to controls, PwMS displayed greater postural sway, greater loading asymmetry, and shorter time-to contact (the time it takes the CoP to contact a stability boundary) during quiet standing. Furthermore, they showed, that limiting vision increased limb loading asymmetry during quiet standing and postural instability during backward lean in PwMS.

Kalron (2016) investigated symmetry in GRF in 402 PwMS. Interestingly, the authors found a vertical GRF symmetry index score of only 3.7% (SD = 3.1). It is known that perfect GRF symmetry, indicated by a symmetry index of zero extremely unlikely, even in non-pathological human gait (Herzog et al., 1989). Therefore, the authors concluded that the score of 3.7% is clinically insignificant. While it seems plausible that favoring one limb over the other, as indicated by limb loading or GRF asymmetries, could predispose individuals to imbalances and falls, the current literature demonstrates contradicting results, where some studies demonstrate asymmetries, while others do not.

## Associations of Muscle Function Asymmetries and Limb Loading Asymmetries on Gait and Balance

Similarly, despite preliminary evidence of existing asymmetries in PwMS, their influence on gait and balance seems unclear. Only a few studies investigated correlations between muscle function asymmetries and functional activities such as walking, climbing stairs, a sit-to-stand test or measures of balance and the evidence appears to be contradicting. Out of the few studies that were of correlational nature, most of them used muscle strength (ability to exert maximal force muscular force) and muscle power (ability to generate muscular work per unit of time) to investigate the influence of asymmetries (Chung et al., 2008; Larson et al., 2013; Sandroff et al., 2013; Proessl et al., 2018). Only Chung et al., 2008 investigated the influence of limb loading asymmetries on gait and balance outcome measures.

On the one side, Larson et al. (2013) showed that isometric strength asymmetries in leg extensor muscles (13.8%) were associated with slower walking speed during a 6-min walk test (6 MWT) in PwMS. Furthermore, Sandroff et al. (2013) showed that aerobic capacity and lower limb isometric strength asymmetries (16.7% knee extensors and 17.7% flexors) explained significant amounts of variance in gait and walking characteristics in PwMS. Chung et al. (2008) also showed a positive correlation between knee extensor power asymmetry and subjective measures of fatigue, the time to complete the 25 ft walk test, as well as anterior–posterior CoP variability during a balance task. However, it is important to note that the latter correlations entailed both, healthy controls and PwMS, and that no associations were reported for the individual groups (PwMS and control). No associations were found between limb loading asymmetry, measured by force plates, and knee extensor or dorsiflexor power asymmetry, suggesting that bilateral peak power differences at the knee and ankle are not responsible for differences in limb loading.

On the other side, a recent study by Proessl et al. (2018) aimed to determine whether leg extensor strength asymmetries are associated with walking ability, objective measures of fatigability, or subjective perception of fatigue. Although PwMS exhibited an average knee extensor strength asymmetry of 21.2%, no significant correlation was identified with walking ability, fatigability or measures of fatigue. Similarly, in the previously mentioned study by Kalron (2016) vertical GRF symmetry did also not influence any of the measures of walking ability in 402 PwMS.

## Reasons for a Lack of Clarity Regarding the Impact of Lower Limb Asymmetries in PwMs

One limitation of current studies investigating the influence of lower limb asymmetries in PwMS is their cross-sectional, comparative nature of the design, because it does not imply causality between measures of walking, strength, and gait kinematics. From the current literature it is not possible to determine whether and how muscle or limb loading asymmetries in PwMS impair gait and balance. Although some studies suggest an importance for asymmetries in walking performance, other studies could not report similar findings, prohibiting practitioners from making informed decisions regarding the rehabilitation process of PwMS.

Current studies investigating muscle function asymmetries also demonstrate variability regarding the assessment, including differing muscles as well as symmetry indices. To limit the influence of these variations on findings regarding the influence of lower limb asymmetries on gait and balance in PwMS, we propose a unified approach for measures of strength (isometric, isotonic, isokinetic) and muscle activity via metabolic imaging in combination with electromyography to evaluate neuromuscular imbalances. A limitation of many studies is the use of force plates (i.e., static posturography) as the only measure of balance. Balance is a combination of many motor and sensory processes that effect postural stability (Chung et al., 2008). Multidimensional measures of balance should be added in future studies in addition to static posturography, for example, dynamic posturography (e.g., Sensory Organization Test), clinical balance assessments (e.g., Berg Balance Test). Furthermore, potential confounding factors such as symptomatic fatigue should be controlled. These measures are capable of examining physiologic variables hypothesized to be responsible for impaired gait and balance caused by muscle asymmetries in PwMS.

If walking performance is under investigation, due to its static nature, maximal isometric strength may not be as good of a predictor as isokinetic or isotonic movements (Broekmans et al., 2013). In the aging literature, for instance, lower extremity power is suggested to be a better predictor of physical function than strength (Bean et al., 2002, 2003). Future research should thus consider power rather than strength as a determinant of muscle function asymmetry in PwMS.

When characterizing muscle function asymmetries in PwMS, it is also critical to use both, a valid functional test as well as measure of muscle asymmetry. While there are limited data detailing differences in functional tests in PwMS, the choice of task can be made on the basis of MS-specific neurophysiological knowledge and data resulting from tasks in healthy people.

Current studies lack high external validity, relying heavily on laboratory tests to determine functional abilities, but failing to address the consequences associated with alterations in these tests. For example, despite investigations of asymmetries on balance and postural control, no study to date has investigated the association between asymmetries in muscle force/power with number of falls in PwMS. However, to differentiate functionally relevant from irrelevant asymmetries as alluded to above, these studies are highly needed and should be conducted in the future.

Finally, questions arise regarding current testing batteries to determine asymmetries in PwMS. Most laboratories currently only rely on a single measure to assess asymmetries and fail to paint a more comprehensive picture including multiple assessments, such as EMG activity, strength, and power, or also sensory function. As the demyelination associated with MS may not only slow efferent signals into the periphery but also afferent feedback to the CNS, including measures of sensory function may provide additional information current testing batteries are lacking. The ability to maintain postural control, for instance, is suggested to be highly influenced by proprioceptive feedback (Fling et al., 2014; Gandolfi et al., 2014) and PwMS have previously demonstrated sensory and proprioceptive impairments. As a consequence, it seems plausible that the inability to appropriately interpret and integrate sensory feedback could contribute to the impaired walking ability that has been reported in PwMS. Therefore, measures of sensory function, such as proprioception, and multiple assessments of muscle function should be included in future testing batteries to more appropriately assess muscle function asymmetries in PwMS.

## Future Directions and Recommendations

Given some of the potential reasons for a lack of clarity regarding the impact of lower limb asymmetry on gait and balance in PwMS, future studies may rather measure power than strength when assessing lower limb asymmetries. In addition, we suggest considering the use of a variety of functional tasks, such as walking and stair climbing. Also, measures of sensory function should be incorporated in future batteries to determine the impact of lower limb asymmetries in PwMS. It is also important to note that majority of PwMS in the references studies were diagnosed with remitting MS except the studies by Chung et al. (2008),Kalron et al. (2011), and Sandroff et al. (2013) which have not reported information regarding MS classification. The effects of muscle and limb loading asymmetries on the different MS types are currently unknown and require future research.

Not only is it unclear whether muscle asymmetry impair gait and balance, but also at which magnitude asymmetries become functionally relevant and whether impairments worsen proportionally to an increase in asymmetries. One-sided muscular adaptation may not always be a pathological finding, as asymmetries may present a necessity to perform tasks of daily living as safe as possible. Unilateral reduction of proprioception, for instance, may result in an increased reliance of the less-affected side during a balance task in order to effectively maintain postural steadiness. Despite limb-loading asymmetries, PwMS in this case would actually use the less-affected leg strategically to maintain functional abilities and reduce the risk for falls. However, if this asymmetry ultimately causes pain or an inability to perform tasks of daily living, it may be considered pathological and would thus require additional treatment. Based on the current literature, these magnitudes of asymmetries have not been found by clinical examinations. Future studies may thus assess the influence of limb loading and muscle function asymmetries on common clinical measures such as the Berg balance scale, the functional gait assessment, the dynamic gait index, or the 6-min walk test.

Once valid measures of muscle asymmetries are identified, the challenge is to identify causal factors. A number of neurophysiological measurements can currently determine adjustments during functional tasks and, importantly, the adjustments during disease progression. These technologies include electromyography, metabolic measures (glucose uptake, blood flow, oxygen consumption and extraction), transcranial direct current stimulation, transcranial magnetic stimulation, structural and functional magnetic resonance imaging, and PET. Given the inconsistency in the current asymmetry literature, it will be increasingly critical to include multiple measures in clinical studies with PwMS when examining perceptions of muscle asymmetries and physiological factors concurrently. For example, Proessl et al. (2018) classified the more- and less-affected side of the body in PwMS using a combination of objective and subjective measures. In their study, asymmetric knee extensor strength was defined as a magnitude of asymmetry exceeding 10%, since other literature considers smaller differences between sides of the body as normal. Indeed, some of the participants did not demonstrate leg muscle asymmetries according to the objective criteria, but subjectively reported experiencing asymmetries. To avoid a Type II error due to a lack of distinction between subjective perceptions of asymmetries and objective measures of asymmetries, we therefore propose to use both assessments in conjunction.

Longitudinal studies, which include various levels of disease severity and duration are needed to determine the causes of muscle asymmetries and whether a greater magnitude of asymmetry may have a more pronounced impact in PwMS.

## Conclusion

Muscle function asymmetries and limb loading asymmetries in PwMS are repeatedly reported. However, the impact of these lower limb asymmetries on functional activities, specifically gait and balance, remains unclear. Reasons for this lack of clarity may include variability regarding the assessment of asymmetries and the determination of asymmetry magnitude through singular measurements only. As a result, we propose a unified approach to assess asymmetries, relying on power rather than strength and incorporating multiple measures, including sensory function, in future testing batteries. In the case of limb loading asymmetries, only one study has investigated their impact on gait and balance, demonstrating the need for future studies in this field. Together with future longitudinal studies incorporating various levels of disease severity and duration, this may then provide more meaningful information regarding the impact of lower limb asymmetries that will allow physical therapists to tailor the rehabilitation process of PwMS.

## Author Contributions

TR and FP contributed to drafting the article and revising it critically for important intellectual content, and approved the final version of the manuscript.

## Conflict of Interest Statement

The authors declare that the research was conducted in the absence of any commercial or financial relationships that could be construed as a potential conflict of interest.
